# Texture and Color Enhancement Imaging for the Kyoto Classification of Gastritis: Evaluation of Visibility and Color Differences

**DOI:** 10.1002/deo2.70297

**Published:** 2026-02-14

**Authors:** Shotaro Oki, Tsutomu Takeda, Yoichi Akazawa, Hiroya Ueyama, Yuji Ikeda, Shin Arii, Takeyasu Sai, Yasuko Uemura, Tomoyo Iwano, Momoko Yamamoto, Ryota Uchida, Hisanori Utsunomiya, Nobuyuki Suzuki, Daiki Abe, Atsushi Ikeda, Noboru Yatagai, Kohei Matsumoto, Kumiko Ueda, Mariko Hojo, Shuko Nojiri, Akihito Nagahara

**Affiliations:** ^1^ Department of Gastroenterology Juntendo University Faculty of Medicine Tokyo Japan; ^2^ Department of Medical Technology Innovation Center Juntendo University School of Medicine Tokyo Japan; ^3^ Department of Pathophysiological Research and Therapeutics for Gastrointestinal Diseases Juntendo University Graduate School of Medicine Tokyo Japan

**Keywords:** color difference, intra‐class correlation coefficient, inter‐rater reliability, Kyoto Classification of Gastritis, texture and color enhancement imaging

## Abstract

**Introduction:**

The Kyoto Classification of Gastritis enables endoscopic assessment of *Helicobacter pylori* (*H. pylori*) infection status and gastric cancer risk. Initially developed for image‐enhanced endoscopy, texture and color enhancement imaging (TXI) allows us to easily distinguish differences in mucosal structure and color. The aim of this study was to evaluate the use of TXI in the visibility of endoscopic findings of gastritis.

**Methods:**

This was a retrospective analysis using prospectively collected endoscopic data from a prospective, single‐center study, in which 220 patients undergoing esophagogastroduodenoscopy were enrolled. Endoscopic images were obtained using both white light imaging (WLI) and TXI (TXI‐1 and TXI‐2). Ten endoscopists (five experts and five trainees) independently evaluated 52 matched image sets, scoring visibility on a 5‐point scale. Inter‐rater reliability was assessed using intraclass correlation coefficients. Objective color analysis using the CIE L*a*b* color space and ΔE* values was also performed for map‐like redness.

**Results:**

TXI‐1 improved visibility for diffuse redness, spotty redness, map‐like redness, patchy redness, atrophic border, red streak, and the regular arrangement of collecting venules. Visibility of intestinal metaplasia was also enhanced by TXI‐1, although to a lesser extent than other findings. TXI‐1 demonstrated “moderate” to “substantial” inter‐rater reliability. Objective colorimetric analysis confirmed significantly greater ΔE* values with TXI‐1 versus WLI for map‐like redness.

**Conclusion:**

TXI‐1 enhances the visibility of key endoscopic features of *H. pylori*–associated gastritis. TXI‐1 may serve as a useful tool for endoscopic assessment of *H. pylori*–associated gastritis.

**Trial Registration:**

The study protocol was registered in the University Hospital Medical Information Network Clinical Trials Registry (UMIN000045323).

## Graphical abstract

Statistically significant differences between the three groups were observed. Furthermore, the ΔE* of texture and color enhancement imaging (TXI)‐1 was significantly greater than that of TXI‐2. Box plots show the median (center line) and interquartile range (box). Whiskers extend to the most extreme values within 1.5×IQR, and values beyond the whiskers are plotted as outliers. * *p* < 0.001.

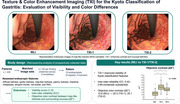

## 1 Introduction

*Helicobacter pylori* (*H. pylori*) infection is widely acknowledged as a significant contributor to the development of gastric cancer, particularly in the context of progressive mucosal atrophy [1]. This association underscores the clinical importance of accurately identifying *H. pylori* infection as well as atrophic gastritis. The Kyoto Classification of Gastritis was introduced in 2014, offering a standardized endoscopic framework to distinguish between current, past, and absent *H. pylori* infection [2, 3].

Texture and Color Enhancement Imaging (TXI; Olympus Medical Systems Corp., Tokyo, Japan), a recently developed modality in image‐enhanced endoscopy (IEE), facilitates clearer visualization of subtle variations in mucosal color and structural patterns [4]. TXI is an imaging technology that enhances endoscopic visualization by integrating three key elements: texture (structural) enhancement, color tone adjustment, and brightness correction. In this system, white light images are separated into texture and base components, along with brightness information, which are then processed and recombined to create TXI mode 2 (TXI‐2). Further enhancement of color tone on TXI‐2 generates TXI mode 1 (TXI‐1), which facilitates the identification of fine structural alterations and subtle color changes, aiding in lesion detection. Recently, TXI has been applied in the upper gastrointestinal field, particularly in studies evaluating mucosal atrophy [5, 6, 7], gastric neoplasms [6, 8, 9, 10, 11, 12, 13, 14, 15, 16, 17, 18, 19, 20], gastric polyps [21], endoscopic assessment of the esophagogastric junction [22, 23, 24], and esophageal lesions [25, 26].

Although TXI has been applied in various gastrointestinal settings, its utility in evaluating gastritis based on the Kyoto Classification of Gastritis remains underexplored. Therefore, we compared the endoscopic visibility of TXI with that of white‐light imaging (WLI) across the Kyoto Classification of Gastritis findings.

## 2 Patients and Methods

### 2.1 Patients

This single‐center prospective study included consecutive patients who underwent esophagogastroduodenoscopy (EGD) with both WLI and TXI between February 2021 and October 2022. Endoscopic findings were evaluated according to the Kyoto Classification of Gastritis.

**FIGURE 1:**
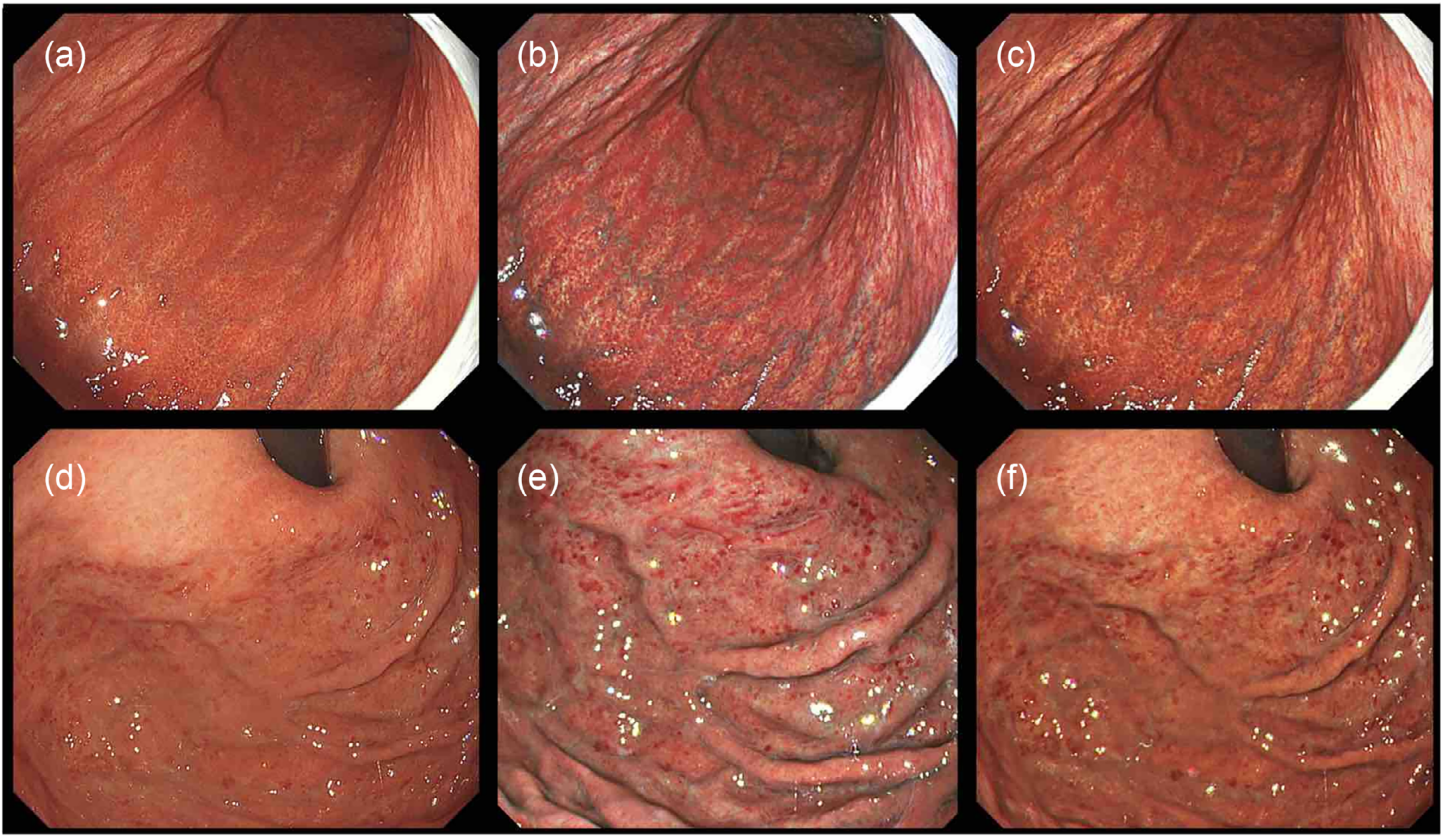
Endoscopic images of stomachs of *Helicobacter pylori* (*H. pylori*)–positive patients using white light, and texture and color enhancement imaging. (a) White light imaging (WLI). The background mucosa of the whole stomach was inflamed, showing diffuse redness and mucosal swelling. (b) Texture and color enhancement imaging mode 1 (TXI‐1). TXI‐1 highlighted the diffuse redness shown. (c) Texture and color enhancement imaging mode 2 (TXI‐2). The diffuse redness is highlighted with TXI‐2. (d) Spotty redness is seen with WLI. (e) TXI‐1 highlighted the spotty redness. (f) The spotty redness is emphasized with TXI‐2, with a similar color to WLI.

**FIGURE 2:**
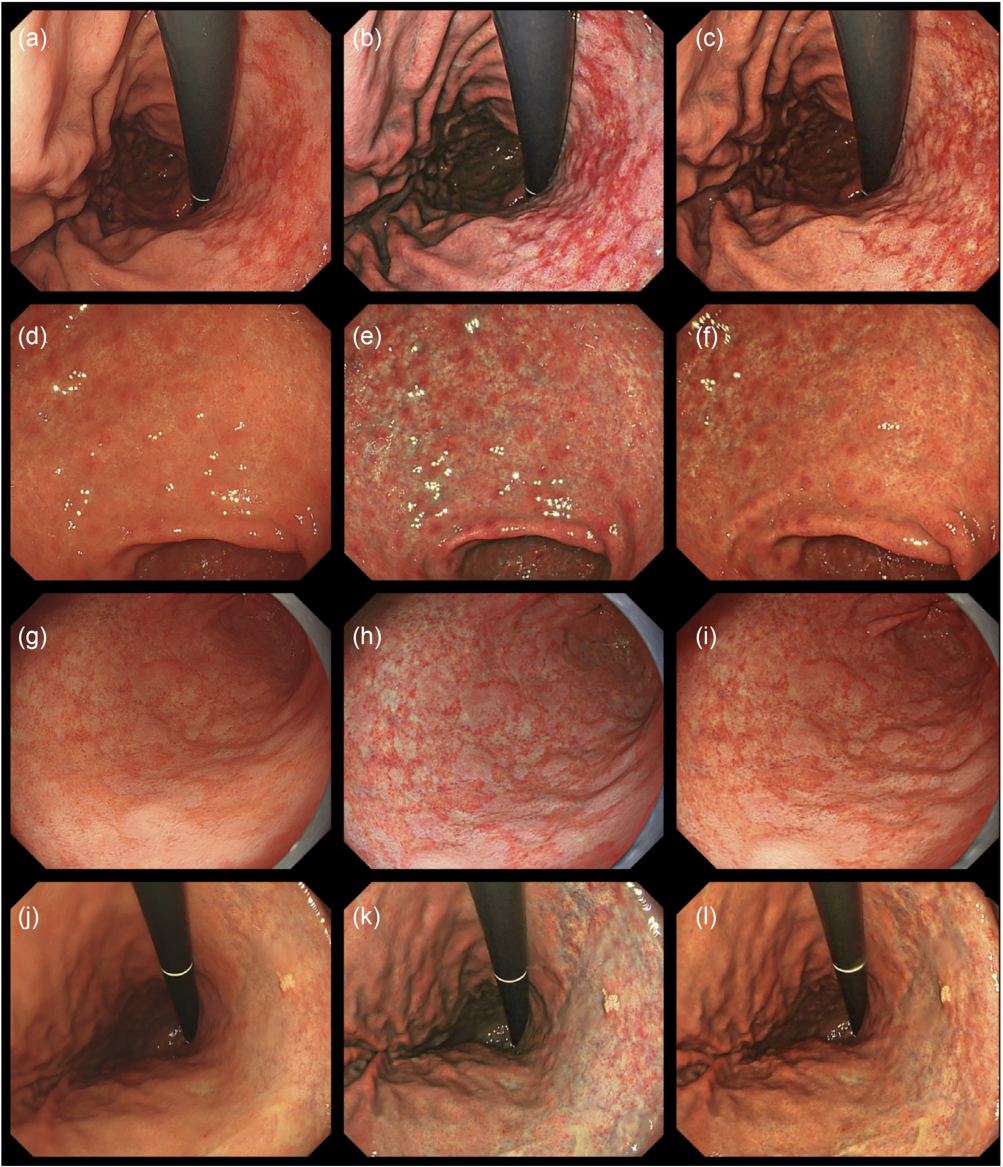
Endoscopic images of stomachs after *Helicobacter pylori* (*H. pylori*) eradication using white light, and texture and color enhancement imaging. (a) White light imaging (WLI). After the eradication of *H. pylori*, a map‐like redness was seen. (b) Texture and color enhancement imaging mode 1 (TXI‐1). The map‐like redness became more defined with TXI‐1. (c) Texture and color enhancement imaging mode 2 (TXI‐2). The map‐like redness is emphasized with TXI‐2. (d) White light imaging (WLI). After *H. pylori* eradication, patchy redness was seen. (e) TXI‐1 highlighted the patchy redness. (f) The patchy redness is emphasized with TXI‐2, with a similar color to WLI. (g) White light imaging (WLI). An intermediate view of the gastric antrum revealed a slightly obscure intestinal metaplasia. (h) TXI‐1. A white flat elevation clearly indicated intestinal metaplasia with TXI‐1. (i) TXI‐2. Intestinal metaplasia was highlighted with TXI‐2. (j) WLI. Atrophic border within the stomach after *H. pylori* eradication. (k) TXI‐1. TXI‐1 led to the atrophic border becoming more defined, and structures were emphasized. (l) TXI‐2. The atrophic border was highlighted with TXI‐2.

**FIGURE 3:**
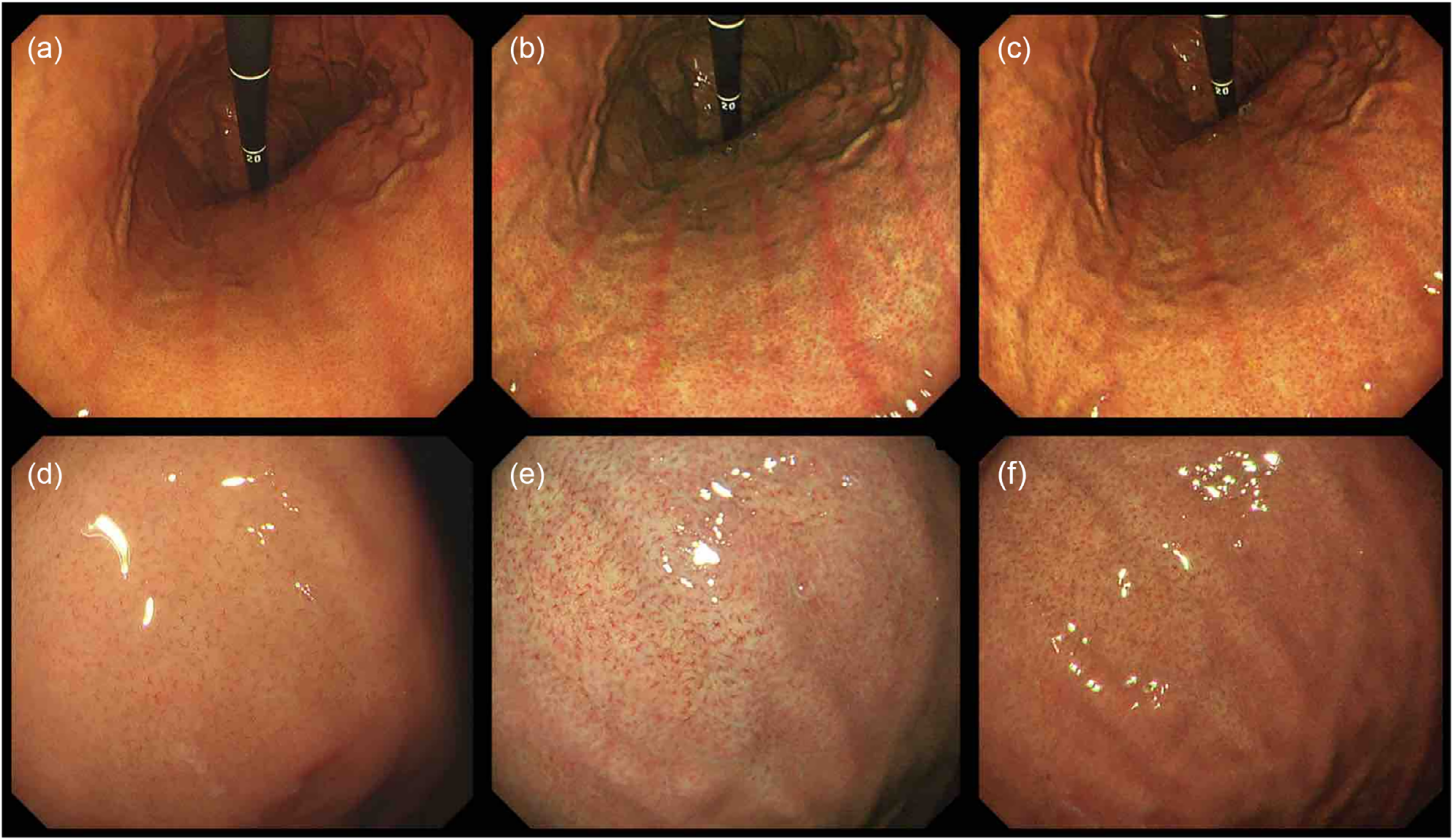
Endoscopic images of red streak and regular arrangement of collecting venules in the stomach of a patient that did not have *Helicobacter pylori* (*H. pylori*) using white light, and texture and color enhancement imaging. (a) White light imaging (WLI). Red streak within a noninfected (*H. pylori*) patient. (b) Texture and color enhancement imaging mode 1 (TXI‐1). TXI‐1 highlighted the red streak. (c) Texture and color enhancement imaging mode 2 (TXI‐2). The red streak was emphasized with TXI‐2. (d) WLI. The regular arrangement of collecting venules (RAC) was seen in uninfected (*H. pylori*) mucosa. (e) TXI‐1. TXI‐1 allowed the RAC to become more defined, and structures were emphasized. f) TXI‐2. RAC was highlighted with TXI‐2.

Inclusion criteria were adults (> 20 years) who underwent EGD with both WLI and TXI and exhibited findings defined in the Kyoto Classification of Gastritis, including diffuse redness, spotty redness, map‐like redness, patchy redness, intestinal metaplasia, atrophic border, red streak, and the regular arrangement of collecting venules (RAC) (Figures 1, 2, 3). Exclusion criteria were prior gastrectomy or esophageal surgery, advanced gastric cancer, active gastric ulcers, poor mucosal visualization due to food residue, and severe systemic disease.

### 2.2 EGD Procedure

Endoscopic examinations were performed using GIF‐H290Z or GIF‐XZ1200 endoscopes with the EVIS X1, CV‐1500 video system (Olympus Medical Systems Corp.). All procedures were conducted by expert endoscopists certified by the Japan Gastroenterological Endoscopy Society. Three experts (Tsutomu Takeda, Yoichi Akazawa, and Hiroya Ueyama) reviewed WLI images and reached a consensus for each finding. Atrophic gastritis was classified according to the Kimura and Takemoto system [27], and reflux esophagitis according to the Los Angeles (LA) classification and its modified version [28, 29, 30]. *H. pylori* infection status was determined by the ^1^ ^3^C‐urea breath test and/or serum antibody testing. A positive result from either test was considered indicative of infection. Successful eradication was defined as a negative ^1^ ^3^C‐urea breath test result 4–8 weeks after therapy.

### 2.3 Evaluation of Visibility of Results According to the Kyoto Classification of Gastritis

This retrospective analysis used prospectively collected WLI and TXI images. Image evaluation was performed retrospectively by expert endoscopists and trainees, all of whom were blinded to patient information. Ten endoscopists—five experts (Kohei Matsumoto, Tsutomu Takeda, Yoichi Akazawa, Noboru Yatagai, and Atsushi Ikeda) and five trainees (Yuji Ikeda, Shin Arii, Takeyasu Sai, Tomoyo Iwano, and Ryota Uchida)—independently assessed 52 image sets (WLI, TXI‐1, and TXI‐2), each derived from a different patient. Experts were defined as endoscopists with more than 10,000 EGD procedures and over 5 years of experience; trainees had less than 5 years of experience. Before scoring, assessors reviewed a brief lecture set to standardize interpretation. Images were shown in random order on a black background using Microsoft PowerPoint 2019 (Microsoft Inc., Redmond, WA, USA). Paired WLI–TXI‐1 or WLI–TXI‐2 images were displayed side by side. All images were anonymized. Visibility was scored on a 5‐point scale: 5 (improved), 4 (somewhat improved), 3 (equivalent), 2 (somewhat decreased), or 1 (decreased). For each finding, expert and trainee scores were summed, with >40 indicating enhanced visibility, 21–39 indicating equivalence, and <20 indicating visibility.

### 2.4 Objective Evaluation in Color Difference

**FIGURE 4:**
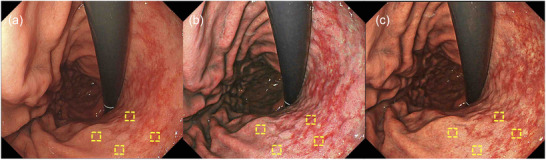
Representative endoscopic images using (a) white light imaging (WLI), (b) texture and color enhancement imaging mode 1 (TXI‐1), and (c) texture and color enhancement imaging mode 2 (TXI‐2) with region of interests (ROIs, 30×30 pixels). Regions of interest (ROIs) were made on the map‐like redness of the mucosa and surrounding gastric mucosa at identical locations in all three images of a specific lesion at two locations each.

Objective evaluation was performed by analyzing L* a* b* color values (L* = light/dark, a* = red/green, b* = yellow/blue) in a CIE LAB color space [31] with Adobe Photoshop CC 2019 as published previously [32, 33]. A region of interest (ROI) measuring 30 × 30 pixels was placed on both the map‐like redness area and the surrounding gastric mucosa (Figure 4).

In our previous color analyses [33, 34, 35], ROI size was selected according to lesion extent and mucosal homogeneity: larger ROIs (400 × 400 pixels) were used for uniform mucosa, whereas smaller ROIs (20 × 20 pixels for localized reflux esophagitis and 40 × 40 pixels for Barrett's mucosa) were applied to localized lesions to avoid contamination. Because map‐like redness occupies an intermediate area with local heterogeneity, a standardized 30 × 30 ROI was used to balance averaging and exclusion of adjacent structures.

For each lesion, ROIs were placed at identical locations across WLI, TXI‐1, and TXI‐2, with two ROIs selected per lesion. Average color values (L, a, b) within each ROI were obtained from the Histogram panel, where L, a, and b represent Lab color units. These values were then converted to CIE LAB color values using the following formulas: L* = (L / 256) × 100, a* = a − 128, and b* = b − 128 [36, 37]. Color differences (ΔE*) were calculated as ΔE* = [(ΔL*)^2^ + (Δa*)^2^ + (Δb*)^2^]^1^^/^^2^ to quantitatively compare modalities.

### 2.5 Ethics

This study was approved by the Ethics Committee of Juntendo University Hospital (Approval No. 20–347) and conducted in accordance with the Declaration of Helsinki. All participants provided their informed consent in writing. This investigation was registered with the University Hospital Medical Information Network (UMIN; #UMIN000045323).

### 2.6 Statistical Analysis

Differences in visibility scores between trainees and experts were assessed using the Mann–Whitney U test. Pairwise comparisons between imaging modes (WLI vs. TXI‐1, WLI vs. TXI‐2, and TXI‐1 vs. TXI‐2) were performed using the two‐sided Wilcoxon signed‐rank test. Differences in L*, a*, b*, and ΔE* values were also analyzed using the Wilcoxon test. A p‐value of < 0.05 was considered statistically significant.

Inter‐rater reliability was evaluated using intra‐class correlation coefficients (ICCs) with 95% confidence intervals (CIs). A two‐way random‐effects model with absolute agreement was used. Such ICCs are widely used to assess the consistency among two or more raters, particularly for ordinal, interval, or ratio‐scale variables [38]. Inter‐rater reliability was interpreted as follows: “perfect” for an ICC = 1.0, “excellent” for ICC >0.81, “substantial” for ICC 0.61–0.80, “moderate” for ICC 0.41–0.60, “fair” for ICC 0.21–0.40, and “slight” for ICC <0.20 [39, 40]. SPSS (version 28.0; IBM, Chicago, IL, USA) was utilized in statistical analyses. To ensure sufficient power for reliability estimation, approximately 52 image sets were required based on standard ICC sample size calculations [41].

## 3 Results

### 3.1 Patient Characteristics

The characteristics of participants are outlined (Table 1). A total of 220 patients were ultimately included to obtain 52 complete image sets for each endoscopic finding, as individual patients did not exhibit all findings. Out of 220 patients, 109 were female, and 111 were male, with an average age of 65.3 years (range: 20–90). Of these patients, 78 were undergoing therapy with a proton pump inhibitor or potassium competitive acid blocker, and 31 patients were taking non‐steroidal anti‐inflammatory drugs. Forty‐nine patients had an *H. pylori* infection, 75 remained uninfected, and 96 had a past history of infection and treatment. Of all patients, 146 had atrophic gastritis (closed type: 73, open type: 73) (Table 1).

**TABLE 1:** Baseline characteristics (*n* = 220).

Characteristics	Value
Sex (males:females)	111:109
Age in years, mean ± SD (range)	65.3 ± 14.3 (20–90)
Atrophic gastritis	C‐0: 74, C‐1–3: 73, O‐1–3: 73
*H. pylori*	negative: 75, positive: 49, post‐eradication: 96
Hiatus hernia	none: 190, present: 30
Reflux esophagitis	none: 179, LA‐M: 20, LA‐A: 17, LA‐B: 3, LA‐C: 1
Antacid therapy	None: 139, PPI/PCAB: 78, H2 blocker: 3
NSAID use	No: 189, Yes: 31

Abbreviations: C, closed, NSAID, non‐steroidal anti‐inflammatory drug, O, open; PCAB, potassium competitive acid blocker; PPI, proton pump inhibitor; SD, standard deviation.

### 3.2 Visibility Scores

Visibility using TXI‐1 and TXI‐2 was compared with WLI in trainees, experts, and all endoscopists. The total visibility scores of all endoscopists are shown in Table 2. A significantly higher visibility score was found among experts for patchy redness, red streak, and RAC in TXI‐1 compared to trainees. Compared to experts, trainees displayed a higher visibility score that was significant for map‐like redness in TXI‐1 and for diffuse, spotty, and map‐like redness in TXI‐2. TXI‐1 was associated with a significantly higher visibility score in all endoscopic findings for all endoscopists compared to TXI‐2. Table 3 shows the evaluation of TXI‐1 and TXI‐2 for visibility and inter‐rater reliability by endoscopists. Improved visibility scores for TXI‐1 compared with WLI were evident in all endoscopists as follows: 82.7% (43/52) for diffuse redness, 98.1% (51/52) for spotty redness, 88.5% (46/52) for map‐like redness, 75.0% (39/52) for patchy redness, 28.8% (15/52) for intestinal metaplasia, 96.2% (50/52) for an atrophic border, 61.5% (32/52) for red streak, and 98.1% (51/52) for RAC. In contrast, TXI‐2 showed lower rates of improved visibility compared with TXI‐1 across all findings. No findings showed decreased visibility in any participant for either TXI mode.

**TABLE 2:** Visibility scores of experts, trainees, and all endoscopists (mean ± SD).

	Trainees (*n*: 5)	Experts (*n*: 5)	Trainees versus experts	All (*n*: 10)	Mode 1 versus mode 2
Endoscopic findings	Total score	Total score	*p‐*Value	Total score	*p‐*Value
Diffuse redness					< 0.001
TXI‐1	20.9 ± 1.3	21.0 ± 1.6	0.74	41.9 ± 2.2	
TXI‐2	19.5 ± 1.6	18.5 ± 1.5	< 0.01	38.0 ± 2.3	
Spotty redness					< 0.001
TXI‐1	21.9 ± 1.3	21.5 ± 1.8	0.17	43.4 ± 2.5	
TXI‐2	20.6 ± 1.3	19.8 ± 1.6	< 0.01	40.4 ± 2.3	
Map‐like redness					< 0.001
TXI‐1	21.4 ± 1.2	20.6 ± 1.6	< 0.01	42.0 ± 2.2	
TXI‐2	19.6 ± 1.4	18.9 ± 1.3	0.02	38.5 ± 2.3	
Patchy redness					< 0.001
TXI‐1	20.3 ± 1.3	21.1 ± 1.5	0.02	41.4 ± 2.2	
TXI‐2	19.5 ± 1.6	19.2 ± 1.7	0.44	38.7 ± 2.7	
Intestinal metaplasia					< 0.001
TXI‐1	19.2 ± 1.5	18.9 ± 2.0	0.45	38.1 ± 2.9	
TXI‐2	17.7 ± 1.4	18.1 ± 1.9	0.21	35.9 ± 2.8	
Atrophic border					< 0.001
TXI‐1	21.6 ± 1.4	21.9 ± 1.5	0.25	43.4 ± 2.4	
TXI‐2	20.5 ± 1.6	20.8 ± 1.3	0.31	41.3 ± 2.5	
Red streak					< 0.001
TXI‐1	19.1 ± 1.2	20.8 ± 1.4	< 0.01	39.9 ± 2.1	
TXI‐2	19.2 ± 1.7	18.9 ± 1.4	0.34	38.1 ± 2.7	
RAC					< 0.001
TXI‐1	21.7 ± 1.3	22.3 ± 1.1	< 0.01	44.0 ± 1.8	
TXI‐2	21.1 ± 1.2	21.4 ± 1.3	0.17	42.5 ± 2.0	

Abbreviations: RAC, regular arrangement of collecting venules; SD, standard deviation; TXI, texture and color enhancement imaging.

**TABLE 3:** Evaluation of texture and color enhancement imaging (TXI) mode 1 and mode 2 for visibility and inter‐rater reliability for all endoscopists (52 cases for each finding).

	TXI‐1			TXI‐2		
Endoscopic finding	Improved visibility (%)	Equivalent visibility (%)	ICC	Improved visibility (%)	Equivalent visibility (%)	ICC
Diffuse redness	43 (82.7)	9 (17.3)	0.48 (0.24–0.67)	15 (28.8)	37 (71.2)	0.45 (0.19–0.64)
Spotty redness	51 (98.1)	1 (1.9)	0.59 (0.41–0.74)	36 (69.2)	16 (30.8)	0.52 (0.31–0.69)
Map‐like redness	46 (88.5)	6 (11.5)	0.43 (0.16–0.63)	17 (32.7)	35 (67.3)	0.47 (0.23–0.66)
Patchy redness	39 (75.0)	13 (25.0)	0.51 (0.28–0.68)	21 (40.4)	31 (59.6)	0.61 (0.43–0.75)
Intestinal metaplasia	15 (28.8)	37 (71.2)	0.68 (0.53–0.79)	8 (15.4)	44 (84.6)	0.67 (0.52–0.79)
Atrophic border	50 (96.2)	2 (3.8)	0.66 (0.51–0.78)	41 (78.8)	11 (21.2)	0.67 (0.52–0.79)
Red streak	32 (61.5)	20 (38.5)	0.52 (0.30–0.70)	18 (34.6)	34 (65.4)	0.69 (0.55–0.80)
RAC	51 (98.1)	1 (1.9)	0.42 (0.16–0.63)	48 (92.3)	4 (7.7)	0.44 (0.18–0.64)

Decreased visibility was not included in the table because all cases were 0% in both TXI modes.

Abbreviations: ICC, intra‐class correlation coefficient (95% confidence interval), RAC, regular arrangement of collecting venules, TXI, texture and color enhancement imaging.

### 3.3 Inter‐Rater Reliability

Intra‐class correlation coefficients (ICCs) for inter‐rater reliability were as follows: 0.48 for diffuse redness, 0.59 for spotty redness, 0.43 for map‐like redness, 0.51 for patchy redness, 0.68 for intestinal metaplasia, 0.66 for atrophic border, 0.52 for red streak, and 0.42 for RAC. For both TXI‐1 and TXI‐2, the inter‐rater reliability ranged from “moderate” to “substantial,” indicating acceptable consistency among endoscopists when assessing results according to the Kyoto Classification of Gastritis (Table 3).

### 3.4 Objective Evaluation

Figure 4 shows representative images of map‐like redness for an ROI. L* a* b* color values of the map‐like redness mucosa, and surrounding gastric mucosa were calculated (Table 4). For map‐like redness, TXI‐1 showed significantly higher a* (*p* = 0.03) and lower b* values (*p* < 0.01) than WLI, with no significant difference in L* values (*p* = 0.11). TXI‐2 demonstrated a significant increase in a* values compared with WLI (*p* < 0.01), while differences in L* (*p* = 0.68) or b* values (*p* = 0.26) were not significant. Between TXI‐1 and TXI‐2, only the b* value showed a significant difference (*p* < 0.01).

**TABLE 4:** Objective evaluations using L*, a*, b* color values and comparison of values in white‐light imaging (WLI), texture and color enhancement imaging (TXI‐1), and TXI‐2.

					*p*‐Value		
	L*, a*, b* values	WLI	TXI‐1	TXI‐2	WLI versus TXI‐1	WLI versus TXI‐2	TXI‐1 versus TXI‐2
Map‐like redness	L* a* b*	49.8 (8.0) 47.8 (7.9) 41.5 (4.0)	52.3 (7.9) 50.3 (9.9) 35.5 (3.8)	50.3 (7.4) 51.0 (7.3) 42.0 (3.9)	0.02 < 0.01 < 0.01	0.55 < 0.01 0.19	0.12 0.82 < 0.01
Gastric mucosa	L* a* b*	60.0 (7.3) 39.3 (6.3) 40.8 (4.3)	68.0 (13.0) 32.8 (10.0) 30.0 (6.0)	65.3 (7.9) 37.0 (7.4) 41.5 (5.8)	< 0.01 < 0.01 < 0.01	< 0.01 < 0.01 0.48	0.16 < 0.01 < 0.01

Abbreviations: SD, standard deviation; TXI, texture and color enhancement imaging; WLI, white light imaging, median (IQR).

**FIGURE 5:**
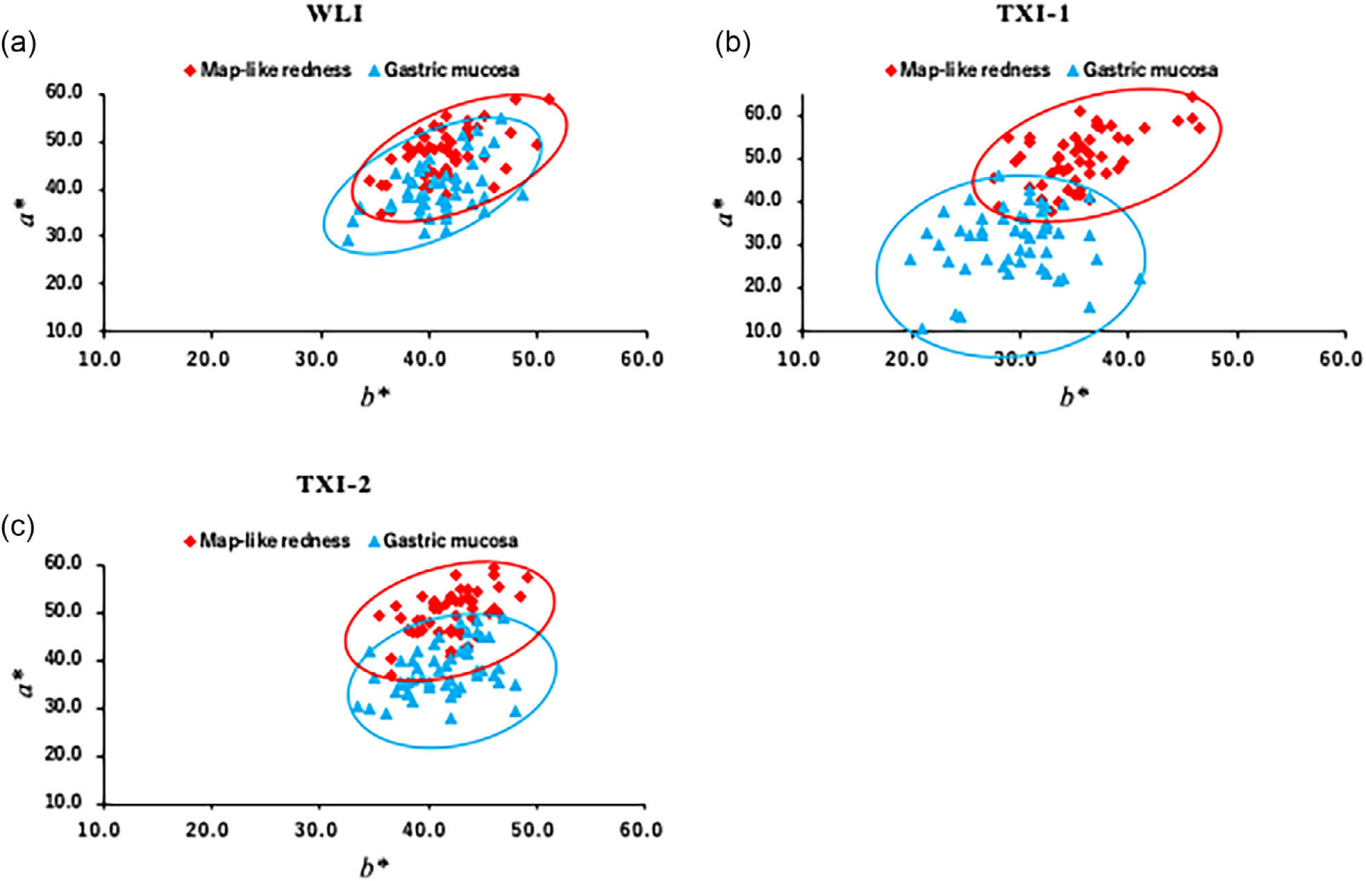
A scatter plot of a*b* color values in CIE LAB. (a) Using white light imaging (WLI) resulted in many overlaps in color distribution between each site. (b) For TXI, color separation and a reduced overlap of color distribution were observed. (c) For TXI‐2, color distribution overlapped between sites.

For the surrounding gastric mucosa, TXI‐1 resulted in significantly higher L* values (*p* < 0.01), and significantly lower a* (*p* < 0.01) and b* values (*p* < 0.01) compared to WLI, indicating an overall enhancement of brightness and color differentiation. TXI‐2 showed a significant difference in a* (*p* = 0.03), whereas b* (*p* = 0.42) did not differ significantly from WLI. A scatter plot was made of a*b* color values in CIE LAB and is shown in Figure 5.

**FIGURE 6:**
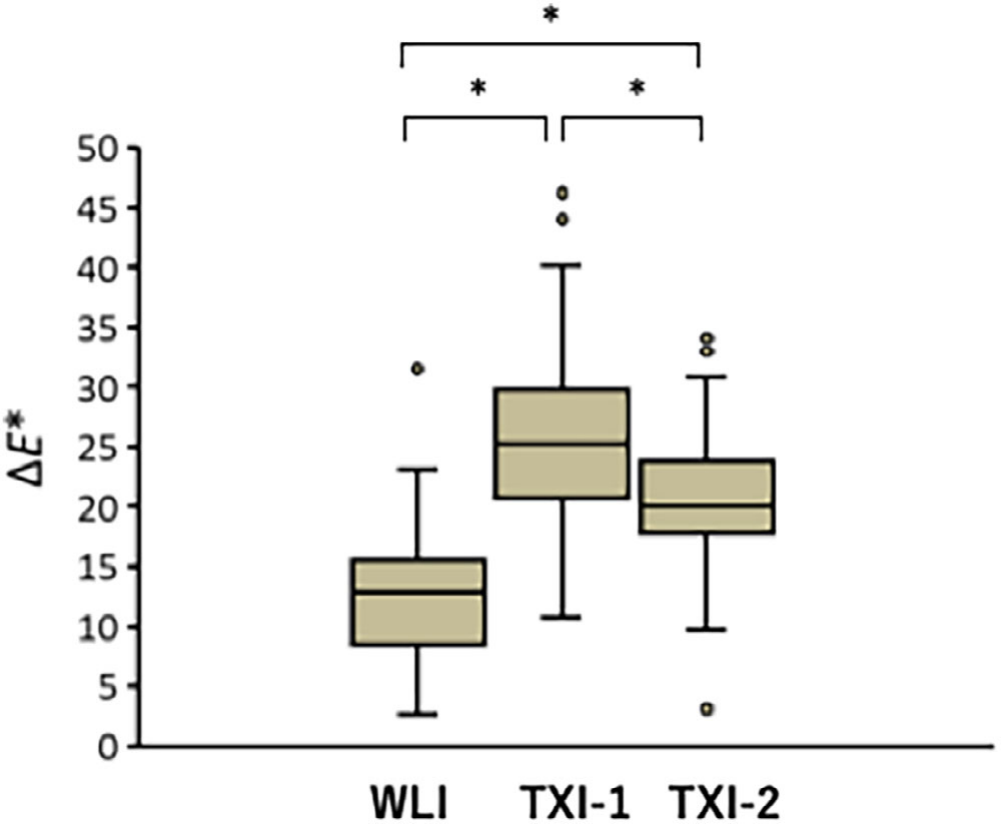
Box plots of the color difference (ΔE*) based on L* a* b* color spaces between the surrounding gastric mucosa and map‐like redness.

Color differences (ΔE*) when comparing map‐like redness and the surrounding gastric mucosa were determined using L*a*b* values in the CIE color space (Table 5). The distribution of ΔE* for map‐like redness across WLI, TXI‐1, and TXI‐2 is shown in Figure 6. ΔE* values were significantly greater for TXI‐1 compared to WLI (*p* < 0.001), indicating enhanced color contrast between the lesion and background. TXI‐2 also showed a significantly higher ΔE* than WLI (*p* < 0.001), although the difference was less than that observed with TXI‐1. Furthermore, the ΔE* of TXI‐1 was significantly greater than that of TXI‐2 (*p* < 0.001), suggesting superior color discrimination with TXI‐1.

**TABLE 5:** Color differences in the CIE L* a* b* color space system between regions of interest (ROIs) and comparisons of values in each imaging mode.

					*p*‐Value		
	L*a*b* values	WLI	TXI‐1	TXI‐2	WLI versus TXI‐1	WLI versus TXI‐2	TXI‐1 versus TXI‐2
Map‐like redness versus Gastric mucosa	ΔE*	12.8 (7.0)	25.3 (9.1)	20.1 (6.1)	< 0.001	< 0.001	< 0.001

Abbreviations: ROI, region of interest; SD, standard deviation; TXI, texture and color enhancement imaging, median (IQR).

## 4 Discussion

In this prospective clinical study, using the Kyoto Classification of Gastritis, we investigated whether TXI improves the visibility of endoscopic results compared to those of WLI. Our results demonstrated that TXI—particularly TXI mode 1 (TXI‐1)—significantly enhanced the visibility of various gastritis‐related findings, including diffuse redness, spotty redness, map‐like redness, patchy redness, atrophic border, intestinal metaplasia, red streak, and RAC. This is the first study to comprehensively evaluate TXI for endoscopic findings defined in the Kyoto Classification of Gastritis.

Several recent investigations corroborate the present results. Ishikawa et al. showed that TXI sharpens gastric mucosal atrophy and neoplasm borders [6], while Sugimoto et al. demonstrated that high‐vision transnasal endoscopy plus TXI (with narrow‐band imaging) improves gastritis detection, particularly in *H. pylori*–positive patients [5]. Kitagawa et al. likewise reported that TXI accentuates subtle mucosal changes associated with *H. pylori* gastritis [7]. In agreement, our data confirm that TXI‐1 elevates the visibility of diffuse and spotty redness, map‐like redness, and the atrophic border, and extends these observations with detailed, lesion‐specific colorimetric analyses. In addition, TXI‐1 also improved the visibility of red streak and RAC, indicating that it may be useful not only for evaluating *H. pylori*‐associated gastritis but also for assessing *H. pylori*–negative gastric mucosa.

Regarding inter‐rater reliability, TXI‐1 showed “moderate” to “substantial” agreement across most findings among both experts and trainees. TXI‐2 showed slightly lower but still acceptable reliability. These results indicate that TXI can provide consistent image interpretation across varying levels of endoscopic experience.

Furthermore, to provide an objective assessment of visibility, colorimetric analysis was conducted using the CIE L*a*b* color space and ΔE* values. TXI‐1 significantly enhanced the color difference between map‐like redness and the surrounding mucosa compared with WLI, suggesting improved contrast and visibility. This quantitative data supports the visual scoring results and emphasizes the utility of TXI‐1 in differentiating subtle mucosal changes that may be challenging to detect under conventional imaging.

To date, linked color imaging (LCI) has also been investigated for its utility in evaluating *H. pylori*–associated gastritis. LCI combines short‐wavelength light with white light, which enhances the contrast between reddish lesions and paler reddish lesions, thereby improving overall color differentiation in gastric mucosa. It also excels in detecting intestinal metaplasia through the visualization of a characteristic lavender color [42]. In our study, TXI‐1 showed relatively lower visibility scores for intestinal metaplasia, which may reflect differences in color enhancement mechanisms between TXI and LCI. Whereas LCI provides distinct wavelength‐based color separation, TXI mainly emphasizes texture enhancement, color tone adjustment, and brightness correction; particularly in TXI Mode 2, the chromatic enhancement remains closer to WLI. These differences may account for the more limited improvement in the visibility of intestinal metaplasia with TXI. Meanwhile, LCI has also been reported to enhance the visibility of diffuse redness, thereby aiding in the diagnosis of active *H. pylori* infection [43]. In a similar manner, TXI‐1 significantly improved the visibility of diffuse redness in our study, suggesting its potential utility in assessing active *H. pylori*–associated gastritis. Our previous study showed that LCI enhanced the visibility and inter‐rater agreement of map‐like and patchy redness, particularly among less experienced endoscopists [44]. In the present study, TXI‐1 also increased the visibility of map‐like redness for trainees in a comparison with experts, indicating its potential value in detecting post‐eradication changes. However, the variable morphology and faint color tone of patchy and map‐like redness may still contribute to inter‐observer variability, particularly among trainees.

Other image‐enhanced endoscopy modalities, such as NBI and BLI, primarily enhance microvascular and surface pattern using short‐wavelength light [45, 46, 47, 48]. These modalities are particularly effective for evaluating lesions in which vascular or surface patterns play a key diagnostic role, such as early gastric neoplasms or inflammatory mucosal changes. In contrast, TXI improves visibility through a different mechanism by emphasizing texture enhancement, color tone adjustment, and brightness correction while preserving a natural mucosal appearance [4]. Because many Kyoto Classification findings —such as diffuse redness, spotty redness, map‐like redness, and atrophic borders—are characterized mainly by broad mucosal color and texture changes rather than vascular alterations, TXI may offer advantages over conventional short‐wavelength light technologies in this context. Thus, TXI serves as a complementary modality to existing image‐enhanced techniques and is particularly suitable for routine assessment of gastritis based on the Kyoto Classification.

Differences between experts and trainees likely reflect the influence of endoscopic experience on image interpretation. Experts may have been better able to utilize subtle texture and brightness enhancements. In contrast, trainees may require additional training to fully benefit from image‐enhanced endoscopy.

Our study had several limitations. First, it was conducted at a single center with a relatively small sample size. Second, observer bias cannot be excluded because visibility was assessed subjectively using still images. Third, whether histological diagnosis and visibility were related was not examined. In addition, endoscopic findings related to *H. pylori* infection may vary by region [49], which was not examined in this study. Furthermore, objective color analysis was based on selected regions of interest rather than whole‐image evaluation, which may limit direct correspondence with the color differences perceived by endoscopists. In the future, a larger prospective study involving histological examinations and the use of endoscopic video images is needed to confirm our findings.

In conclusion, TXI‐1 substantially enhances the visibility and interpretability of key endoscopic findings defined in the Kyoto Classification of Gastritis. It improves both lesion‐specific contrast and inter‐rater agreement, particularly for inflammatory and atrophic changes. TXI‐1 may serve as a valuable tool in the comprehensive assessment of *H. pylori*–related gastritis and gastric cancer risk during routine endoscopic screening.

## Author Contributions

**Shotaro Oki**: formal Analysis (lead); investigation (lead); writing – original draft preparation (lead); writing – review and editing (lead). **Tsutomu Takeda**: conceptualization (lead); formal analysis (lead); investigation (lead); writing – original draft preparation (lead); writing – review and editing (lead). **Yoichi Akazawa**: methodology (lead); writing – review and editing (equal). **Hiroya Ueyama**: methodology (lead); writing – review and editing (equal). **Yuji Ikeda**: investigation (equal). **Shin Arii**: investigation (equal). **Takeyasu Sai**: investigation (equal). **Yasuko Uemura**: investigation (equal). **Tomoyo Iwano**: investigation (equal). **Momoko Yamamoto**: investigation (equal). **Ryota Uchida**: investigation (equal). **Hisanori Utsunomiya**: investigation (equal). **Nobuyuki Suzuki**: investigation (equal). **Daiki Abe**: investigation (equal). **Atsushi Ikeda**: investigation (equal). **Noboru Yatagai**: investigation (equal). **Kohei Matsumoto**: investigation (equal). **Kumiko Ueda**: writing – review and editing (supporting). **Mariko Hojo**: writing – review and editing (supporting). **Shuko Nojiri**: data curation (lead). **Akihito Nagahara**: conceptualization (lead); methodology (lead); project administration (lead); supervision (lead); writing – review and editing (lead).

## Funding

The authors received no specific funding for this work.

## Ethics Statement

Approval of the research protocol by an Institutional Reviewer Board: The present study was approved by the ethics committee of Juntendo University Hospital (No. 20–347) and was performed according to the tenets of the Declaration of Helsinki.

## Consent

All patients provided written informed consent to participate in this study.

## Conflicts of Interest

Tsutomu Takeda and Akihito Nagahara were supported by Olympus Co., on loan of equipment (EVIS X1:CV‐1500).
